# Better Correlation of Cognitive Function to White Matter Integrity than to Blood Supply in Subjects with Leukoaraiosis

**DOI:** 10.3389/fnagi.2017.00185

**Published:** 2017-06-12

**Authors:** Genlong Zhong, Ruiting Zhang, Yerfan Jiaerken, Xinfeng Yu, Ying Zhou, Chang Liu, Longting Lin, Lusha Tong, Min Lou

**Affiliations:** ^1^Department of Neurology, School of Medicine, The Second Affiliated Hospital of Zhejiang UniversityHangzhou, China; ^2^Department of Radiology, School of Medicine, The Second Affiliated Hospital of Zhejiang UniversityHangzhou, China; ^3^The School of Medicine and Public Health, University of Newcastle, NewcastleNSW, Australia

**Keywords:** cerebral blood flow, cognitive function, diffusion tensor imaging, diffusion kurtosis imaging, white matter disease

## Abstract

Leukoaraiosis is associated with increased risk of cognitive impairment, but its pathophysiological pathway is unclear. The aim of the present study was to determine whether brain structural damage or cerebral blood supply better correlated with the global cognitive outcome in subjects with leukoaraiosis. Seventy-five subjects with leukoaraiosis were included in present study, with age ranged from 43 to 85 years, with mean white matter hyperintensities (WMH) volume 30.69 ± 24.35 mL. Among them, 19(25.33%) subjects presented with cerebral microbleeds (CMB) and 40 (53.33%) subjects presented with lacunes. These participants received arterial spin labeling perfusion MRI, diffusion-tensor imaging (DTI) and diffusion Kurtosis imaging. We analyzed the cerebral blood flow (CBF) by dividing the brain tissue into three regions: WMH, normal appearing white matter (NAWM) and cortex. After adjusting for age and gender, the CBF of NAWM was significantly correlated with fractional anisotropy (FA) (*r* = 0.336, *p* = 0.004) and mean diffusion (MD) (*r* = -0.271, *p* = 0.020) of NAWM, while there lacked of association between CBF of cortex and mean kurtosis (MK) of cortex (*r* = -0.015, *p* = 0.912). Meanwhile, both NAWM-FA (*r* = -0.443, *p* < 0.001) and NAWM-MD (*r* = 0.293, *p* = 0.012), as well as cortex-MK (*r* = -0.341, *p* = 0.012) was significantly correlated with WMH volume. Univariate regression analysis demonstrated that global cognitive function was significantly associated with mean FA or MD of both WMH and NAWM, and cortex-CBF, but neither with the cortex-MK, nor the presences of CMB or lacunes. Finally, multiple linear regression analysis revealed that global cognitive function was independently associated with NAWM-FA (standardized β = 0.403, *p* < 0.001) and WMH-FA (Standardized β = 0.211, *p* = 0.017), but not with the cortex-CBF. A model that contained NAWM-FA, WMH-FA and years of education explained 49% of the variance of global cognitive function. Cerebral perfusion status might have a significant impact on the maintenance of white matter integrity in subjects with leukoaraiosis. Global cognitive function was more strongly associated with white matter integrity than with blood supply. DTI parameters, especially FA could serve as a potent imaging indicator for detecting the invisible alteration of white matter integrity and implying its potential cognitive relevance.

## Introduction

Leukoaraiosis, also known as white matter hyperintensities (WMH) of presumed vascular origin, are frequently seen on magnetic resonance imaging (MRI) scans of the brain in the elderly (Wardlaw et al., 2013). WMH has been viewed as an etiology of cognitive impairment and vascular dementia (VaD) (Prins and Scheltens, 2015). However, the association between the burden of WMH and severity of cognitive impairment is controversial. Some studies found that neuropsychological impairment was correlated with cerebral hypoperfusion but not the severity of WMH in patients with small vessel diseases (SVD) (Sabri et al., 1999). Evidence also showed that severe carotid stenosis with hemodynamic impairment would significantly increase the risk of cognitive deterioration in asymptomatic subjects (Balestrini et al., 2013).

The adverse connection between cerebral hypoperfusion and cognitive dysfunction may be mediated by disruption of white matter microstructural integrity, which was found to be associated with cognitive performance based on diffusion tensor imaging (DTI) studies (Nitkunan et al., 2008; Vernooij et al., 2009). Alternatively, cerebral hypoperfusion may directly compromise the brain function by down-regulation of neural metabolism and electrical activity, leading to neuronal adaptive responses (Banerjee et al., 2016; Bordeleau et al., 2016). Besides, cerebral hypoperfusion would accelerate deposition of Aβ-amyloid, contributing to the concurrence of VaD and Alzheimer’s disease (AD) (Zhiyou et al., 2009). Furthermore, recent studies also suggested that cortex alterations along the course of WMH may also contribute to cognitive decline (Peres et al., 2016). Cortex alterations (e.g., Microinfarcts, atrophy) and WMH may share common underlying mechanism such as chronic hypoperfusion. The emerging technique of diffusion kurtosis imaging (DKI) is promising to detect the disruption of cortex microarchitecture and its cognitive relevance as shown in study among multiple sclerosis (MS) patients (Bester et al., 2015).

Currently, it is unclear whether brain structural damage or blood supply condition could better predict the global cognitive outcome in subjects with leukoaraiosis. In present study, we utilized multi-model MRI to assess the cerebral perfusion, white matter and cortex microstructural integrity, as well as the burden of WMH, brain volume, presence of cerebral microbleeds (CMB) and lacunes. Then we investigated their relationship with global cognitive function among a cohort of subjects with leukoaraiosis. It is meaningful to identify potent imaging indicators for global cognitive performance, which would not only favor elucidating the pathophysiological pathway of cognitive impairment, but may also be helpful for assessment of therapeutic efficacy in future interventional trials.

## Materials and Methods

### Study Subjects

This was an investigator-initiated prospective single-center study. Patients with WMH were recruited between January 2014 and April 2016 from our department. We then enrolled those who met all of the following inclusion and none of the exclusion criteria into this study. Inclusion criteria: (1) WMH visible on Fluid-Attenuated-Inversion-Recovery (FLAIR) images; (2) age ≥ 40; (3) without a history of MS, AD, Parkinson’s disease and head trauma; (4) agreed to give written informed consent. Exclusion criteria: (1) patients with secondary causes of white matter lesions, such as immunological, demyelinating, metabolic, toxic, infectious, and other established causes; (2) patients with abnormal brain MRI findings such as space-occupying lesions, head trauma, hemorrhage, or infarction (except for lacunar infarction); (3) patients with poor image quality. The patients admitted for TIA or acute lacunar stroke were recruited at least 3 months after the date of last stroke to avoid acute effects on MRI or neuropsychological findings. We retrieved demographic, clinical and imaging data including age, gender, years of education, and the medical history such as hypertension, diabetes mellitus, hyperlipidemia, and smoking history. Global cognitive function was assessed by Mini-Mental State Examination (MMSE).

### Ethics Statement

All subjects had given written informed consent prior to the study, and the protocols had been approved by the human ethics committee of the Second Affiliated Hospital of Zhejiang University School of Medicine. All clinical investigation has been conducted according to the principles expressed in the Declaration of Helsinki.

### WMH Identification and Volume Measurement

Identification of WMH on FLAIR images was according to “neuroimaging standards for research into small vessel disease” published by Wardlaw et al. (2013). WMH volume was semiautomatically measured (details seen in Image Analysis section).

### Definition and Detection of Cerebral Microbleeds and Lacunes

Cerebral microbleeds were defined as small (≤ 10 mm), homogeneous, round foci of low signal intensity on gradient echo images in cerebellum, brainstem, basal ganglia, white matter, or cortico-subcortical junction, differentiated from vessel flow voids and mineral depositions in the globi pallidi (Greenberg et al., 2009). Lacunes were defined as rounded or ovoid lesions, 3–15 mm of diameter, in the basal ganglia, internal capsule, centrum semiovale, or brainstem, of cerebrospinal fluid (CSF) signal intensity on T1-,T2-weighted imaging and FLAIR, generally with a hyperintense rim on FLAIR and no increased signal on diffusion weighted-imaging (DWI)(Wardlaw et al., 2013).

### MRI Protocol

All subjects underwent multi-model MRI by a 3.0 T MR (MR750, GE Healthcare, United States) scanner using an 8-channel brain phased array coil, including high-resolution three dimensional (3D) sagittal T1-weighted imaging (T1WI), FLAIR, DTI and 3D arterial spin labeling (ASL) perfusion MRI. A subset of subjects also received DKI. For DTI, we performed a single shot, diffusion-weighted spin echo echo-planar imaging sequence. Maximum *b*-value was 1000 s/mm^2^ in 30 non-collinear directions; 1 volume was acquired without diffusion weighting (*b*-value = 0 s/mm^2^). Other parameters of DTI were as follows: repetition time (TR)/echo time (TE) = 8000/80.8 ms, flip angle = 90°, slice thickness = 2 mm without slice gap, matrix size = 128 × 128, field of view (FOV) = 25.6 cm. DKI was acquired using a single-shot, spin–echo planar imaging (EPI) sequence with TR/TE = 5000/95 ms; slice thickness = 2 mm without slice gap; FOV: 256 mm × 256 mm; matrix: 128 × 128; and three diffusion weighting (*b*) values (0, 1000, and 2000 s/mm^2^), with diffusion encoding in 30 directions for every *b*-value. 3D ASL was acquired using spin-echo pulse sequence with TR/TE = 4611/10.5 ms, inversion time (TI) = 1525 ms, flip angle = 111°, slice thickness = 4 mm, matrix = 128 × 128, FOV = 24 cm. High-resolution 3D sagittal T1-weighted imaging (T1WI) was acquired using spoiled gradient echo sequence with TR/TE = 7.3/3.0 ms, TI = 450 ms, flip angle = 8°, slice thickness = 1 mm, matrix = 250 × 250, FOV = 25 cm. Conventional MRI sequences included FLAIR (TR/TE = 8400/152 ms, TI = 2100 ms, flip angle = 90°, slice thickness = 4 mm without slice gap, matrix size = 256 × 256, FOV = 24 cm), T1WI (TR/TE = 1750/25 ms, TI = 780 ms, flip angle = 90°, slice thickness = 4 mm without slice gap, matrix size = 256 × 256, FOV = 24 cm) and DWI (TR/TE = 5000/86 ms, flip angle = 90°, slice thickness = 4 mm without slice gap, matrix size = 256 × 256, FOV = 24 cm, *b*-value = 1000 s/mm^2^ along three orthogonal directions).

### Image Analysis

DTI images were post-processed using FSL^1^ to extract brain, remove bulk motion, and eddy current induced distortions. Then we calculated the parametric maps of fractional anisotropy (FA) and mean diffusivity (MD) with DTIfit command in FSL. DKI images were corrected for brain distortion using the “eddy-current” toolbox in FSL. With Diffusion Kurtosis Estimator (DKE)^2^, mean kurtosis (MK) was calculated on DKI parametric map. The segmentation of gray matter, normal appearing white matter (NAWM) and WMH tissue masks was automatically processed in the native space using 3D T1WI and FLAIR images by lesion segmentation tool (LST) toolbox (Schmidt et al., 2012) in Statistical Parametric Mapping Version 8 (SPM8). The processed WMH, NAWM and gray matter masks were further manually corrected by an experienced neuroradiologist using ITK-SANP software^3^. The steps of manual correction include: (1) removal of non-brain tissue, deep gray matter, brain stem and cerebellum; (2) correction of false segmentation (positives or negatives). After coregistration, the masks of WMH, NAWM and cortex were used to obtain averaged FA, MD, MK and cerebral blood flow (CBF) of corresponding tissues in each participant (as illustrated in **Supplementary Figure S1**). Total brain volume was calculated by taking the sum of the gray matter and white matter probabilistic tissue maps and multiplying this by the voxel volume (1 mm^3^). Intracranial volume (ICV) was calculated as the sum of total brain volume and CSF. WMH volume and brain volume be used in present study were the adjusted volume by ICV.

### Statistical Analysis

All numeric variables were expressed as mean ± SD. The difference between FA, MD, and CBF of WMH and their counterparts of NAWM were compared using paired *t*-tests. The Pearson’s partial correlation between CBF and the metrics of microstructural integrity (FA, MD or MK) were tested, controlling for age and sex. The association of CBF, microstructural integrity, WMH volume, presences of CMB or lacunes with MMSE scores were tested using univariate linear regression model. The association of the variables, whose *P* < 0.1 in above univariate linear regression models, with MMSE score was estimated using the multiple stepwise linear regression model (Model 1). Model 2 included the original variables of model 1, besides age, gender, years of education, brain volume, WMH volume, and presence of CMB or lacunes. A level of *P* < 0.05 was considered statistically significant. All statistical analyses were performed by IBM SPSS Statistics 21.

## Results

### Patient Characteristics

The demographic, clinical and imaging characteristics were demonstrated in **Table 1**. Seventy five subjects with leukoaraiosis were included in present study. The main reasons for admission of those patients were TIA or lacunar ischemic stroke (*n* = 44, 58.67%), dizziness (*n* = 19, 25.33%), cognitive impairment (*n* = 5, 6.67%), headache (*n* = 5, 6.67%), or gait disturbance (*n* = 2, 2.67%). Their age ranged from 43 to 85 years and WMH volume ranged from 1.54 to 120.51 mL, with mean MMSE Score 25.91 ± 3.50. Among them, 19(25.33%) subjects presented with microbleeds and 40 (53.33%) subjects presented with lacunes. To be note, the analysis referring to MK was restricted to a subset of 56 participants receiving DKI.

**Table 1:** Baseline characteristics.

Variable	*N* = 75 (mean ±*SD*) or *n*(%)
Age (*y*)	67.05 ± 9.62
Female%	39 (52.00%)
Years of education (*y*)	6.44 ± 4.75
MMSE score	25.91 ± 3.50
Hypertension	52 (69.33%)
Diabetes mellitus	17 (22.67%)
Hyperlipidemia	19 (25.33%)
Smoking	16 (21.33%)
WMH volume, mL	30.69 ± 24.35
Brain volume, mL	1004.14 ± 40.90
Presence of microbleeds	19 (25.33%)
Presence of lacunes	40 (53.33%)

*MMSE, Mini-Mental State Examination; FA, fractional anisotropy; MD, mean diffusivity; CBF, cerebral blood flow; WMH, white matter hyperintensities; NAWM, normal-appearing white matter*.

### Correlation between Cerebral Blood Flow and Microstructural Integrity

**FIGURE 1:**
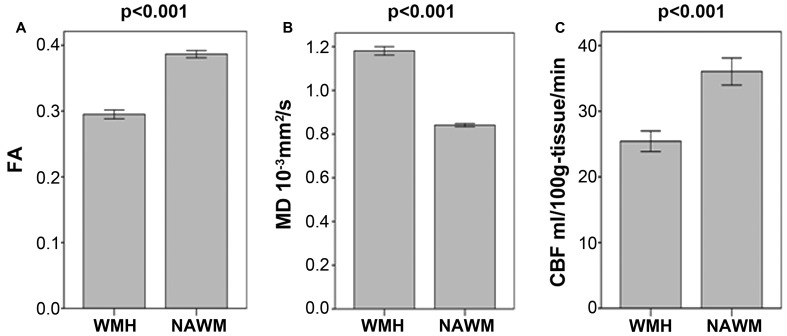
**(A–C)** Comparisons of mean fractional anisotropy (FA), mean diffusion (MD) and cerebral blood flow (CBF) in white matter hyperintensities (WMH) and in their corresponding normal appearing white matter (NAWM).

As illustrated in **Figure 1** and **Table 2**, FA and CBF of WMH were significantly decreased compared to that of NAWM, while MD was significantly increased. After adjusting for age and gender, NAWM-FA (*r* = 0.336, *p* = 0.004) and NAWM-MD (*r* = -0.271, *p* = 0.020) were low and significantly correlated with NAWM-CBF, while there lacked of association between cortex-CBF and cortex-MK (*r* = -0.015, *p* = 0.912) (**Table 3**). Meanwhile, both NAWM-FA (*r* = -0.443, *p* < 0.001) and NAWM-MD (*r* = 0.293, *p* = 0.012), as well as cortex-MK (*r* = -0.341, *p* = 0.012) were low while significantly correlated with WMH volume after adjusting for age and gender.

**Table 2:** The distribution of microstructural integrity and cerebral blood flow of WMH, NAWM and Cortex.

Variable	*N* = 75 (mean ±*SD*)
WMH-FA	0.2949 ± 0.0292
NAWM-FA	0.3865 ± 0.0240
WMH-MD,10^-3^mm^2^/s	1.1809 ± 0.0830
NAWM-MD,10^-3^mm^2^/s	0.8408 ± 0.0326
WMH-CBF,mL/100g-tissue/min	25.43 ± 6.79
NAWM-CBF, mL/100g-tissue/min	36.05 ± 8.97
Cortex-MK	0.5430 ± 0.0982^#^
Cortex-CBF, mL/100g-tissue/min	44.87 ± 11.48

*FA, fractional anisotropy; MD, mean diffusivity; CBF, cerebral blood flow; WMH, white matter hyperintensities; NAWM, normal-appearing white matter; MK, mean kurtosis. ^#^indicates subset analysis*.

**Table 3:** Pearson’s partial correlation between cerebral blood flow and microstructural integrity after adjustment for age and sex.

	WMH-CBF		NAWM-CBF		Cortex-CBF	
	*r*	*P*-value	*r*	*P*-value	*r*	*P*-value
WMH-FA	0.083	0.484	–	–	–	–
WMH-MD	-0.133	0.264	–	–	–	–
NAWM-FA	–	–	0.336	0.004	–	–
NAWM-MD	–	–	-0.271	0.020	–	–
Cortex-MK	–	–	–	–	-0.015	0.912^#^

*FA, fractional anisotropy; MD, mean diffusivity; CBF, cerebral blood flow; WMH, white matter hyperintensities; NAWM, normal-appearing white matter; MK, mean kurtosis. ^#^indicates subset analysis*.

### Relationship between Metrics of Brain Structural Damage, CBF and Global Cognitive Function

Univariate linear regression analysis demonstrated that MMSE score was significantly associated with mean FA or MD of both WMH and NAWM, and CBF of cortex, but not with the MK of cortex (**Table 4**). Nevertheless, MMSE score was not significantly associated with WMH volume (standardized β = -0.125, *p* = 0.284), nor the presences of CMB (standardized β = 0.060, *p* = 0.611) or presences of lacunes (standardized β = -0.048, *p* = 0.082). Correlation between MMSE score and individual imaging parameters were presented in the scatter plots of **Supplementary Figure S2**. Finally, multiple linear regression analysis revealed that global cognitive function was independently associated with NAWM-FA (standardized β = 0.403, *p* < 0.001) and WMH-FA (standardized β = 0.211, *p* = 0.017), but not with cortex-CBF (**Table 5**). A model that contained of NAWM-FA, WMH-FA and years of education explained 49% of the variance of MMSE score.

**Table 4:** The association of microstructural integrity, cerebral blood flow with MMSE score using univariate linear regression model.

Variable	Standardized β	*R*^2^	*P*-value
WMH-FA	0.328	0.108	0.004
NAWM-FA	0.501	0.251	<0.001
WMH-MD	-0.205	0.042	0.078
NAWM-MD	-0.258	0.066	0.026
WMH-CBF	0.184	0.034	0.114
NAWM-CBF	0.207	0.043	0.075
Cortex-CBF	0.248	0.062	0.032
Cortex-MK	0.076	0.006	0.580^#^

*MMSE, Mini-Mental State Examination; FA, fractional anisotropy; MD, mean diffusivity; CBF, cerebral blood flow; WMH, white matter hyperintensities. NAWM, normal-appearing white matter; MK, mean kurtosis. *R*^*2*^ represents the proportion of variation in MMSE score explained by individual variable. ^#^indicates subset analysis*.

**Table 5:** The association of relevant variables ^∗^ with MMSE score using multiple stepwise linear regression model.

	Standardized β	*P*-value	*R*^2^
**Model 1**
WMH-FA	0.266	0.008	0.321
NAWM-FA	0.466	<0.001	
**Model 2**
WMH-FA	0.211	0.017	0.490
NAWM-FA	0.403	<0.001	
years of education	0.421	<0.001	

*FA, fractional anisotropy; WMH, white matter hyperintensities; NAWM, normal-appearing white matter. ^∗^Relevant variables included WMH-FA, NAWM-FA, WMH-MD, NAWM-MD, NAWM-CBF and cortex-CBF in model 1; Additionally, age, gender, years of education, brain volume, WMH volume, and presence of cerebral microbleeds or lacunes were further included in Model 2. *R*^*2*^ represents the proportion of variation in MMSE score explained by all variables in model 1 and model 2*.

## Discussion

For subjects with leukoaraiosis, brain structural damage has been reported to cause their cognitive impairment, whereas evidence also showed the importance of blood supply in affecting their cognitive outcome. Limited studies have been conducted to link the two underlying mechanisms. This study explored the relationship of these two mechanisms and results showed that global cognitive function was more strongly associated with white matter integrity than with blood supply in subjects with leukoaraiosis.

Degradation of the white matter microarchitecture could be detected by changes in measurable DTI parameters, reflecting as decreased FA or increased MD (Moseley, 2002). FA and MD of NAWM were found to be significantly associated with WMH volume in present study, which implied that invisible structural damages were beyond the boundary of WMH lesion. And we observed that FA of both WMH and NAWM were closely correlated with global cognitive performance. And a model that contained FA of WMH, FA of NAWM and years of education could explain nearly half of the variance of MMSE score. This was consistent with previous large population-based study which demonstrated that DTI parameters of both WMH and NAWM were significantly associated with cognitive function, regardless of WMH load and brain atrophy (Vernooij et al., 2009). Study conducted by Nitkunan et al. (2008) also demonstrated that the variance of executive function could largely be explained by a model that contained peak height of FA, brain volume, together with age, gender, and premorbid IQ among 35 patients with SVD.

Meanwhile, our data again showed weak correlation between WMH load and cognitive performance, which could be attributed to the difference of underlying pathological changes both in similar appearing WMH lesions and in NAWM. Postmortem studies also proved that WMH are heterogeneous in terms of histopathology, ranging from slight disentanglement of the matrix to varying degrees of demyelination and axonal loss (Gouw et al., 2011).

Previous study reported that neuropsychological impairment was significantly correlated with cerebral hypoperfusion but not the severity of WMH in patients with SVD (Sabri et al., 1999). Moser et al.’s (2012) study also found that global CBF were significantly associated with global neuropsychological functioning after adjusting for age and sex. Nevertheless, these studies did not take into account the invisible alterations of white mater integrity, which was significantly correlated with the cognitive function in present and other studies. We posited that the previous reported adverse connection between cerebral hypoperfusion and cognitive dysfunction might be mediated by disruption of white matter integrity secondary to chronic hypoperfusion. Supporting this assumption, we found that CBF of WMH was significantly decreased compared to that of NAWM, and CBF of NAWM was significantly correlated with FA of NAWM after adjusting for age and sex. Another study based on PET-CT also showed no association between baseline global CBF and MMSE score among 27 cognitively intact individuals with hypertension and WMH (Kitagawa et al., 2009). However, this study prospectively observed that baseline CBF was linearly associated with cognitive decline assessed by MMSE score over a 3-years period. This paradoxical finding could be explained by that lower baseline CBF leads to accelerated but non-proportionate disruption of white matter integrity, which then contributes to cognitive deterioration.

Diffusion kurtosis imaging allows the measurement of MK, which does not require tissue’s directionality and hence it is applicable for measuring microstructural integrity of cortex. Previous studies have shown that cortical atrophy was associated with the burden of WMH, but little is known about the condition of the tissue not yet lost. We found that cortical MK was inversely associated with WMH volume, while there lacked of association between cortical MK and cortical CBF. This could be pathophysiologically explained by that cortex alterations may be secondary to remote white matter degeneration (e.g., WMH) other than hypoperfusion. Among 34 MS patients, a preliminary study found that decreased MK of cortex was correlated with poor cognitive performance (Bester et al., 2015). By contrast, we found no significant association between MK of cortex and cognitive function, indicating the relative health of the existing cortex with regard to neuronal degeneration. However, the exact pathological substrate responsible for MK changes of cortex has yet to be elucidated.

To the best of our knowledge, few previous work, if any, has investigated the correlation between cerebral perfusion and brain microstructural integrity, as well as their relationship with global cognitive function in subjects with leukoaraiosis. Methodologically, we analyzed the CBF by dividing the brain tissue into three regions: WMH, NAWM and cortex. By contrast, previous studies based on PET-CT could only measure the CBF of regional lobe or whole brain due to its limited spatial resolution. Given the CBF varies greatly in white matter and cortex, mixed quantification of CBF in these territories would compromise its reliability in assessing its relationship with structural metrics and cognitive function. Our findings suggested that DTI parameters, especially FA of NAWM, could serve as a candidate imaging indicator for monitoring alterations of global white matter integrity and implying its cognitive relevance. By contrast, cerebral blood supply is not an ideal indicator for predicting cognitive function in subjects with leukoaraiosis. Though cerebral hypoperfusion has detrimental effect on the integrity of white matter, ischemic tolerance may vary among different individuals. Besides, risk factors other than hypoperfusion may also contribute to the injury of white matter, such as endothelial dysfunction and increased blood brain barrier permeability (Wardlaw, 2010).

Our study also has some limitations. Firstly, the modest sample size may limit its power to reveal the relationship between cognitive function and imaging parameters. Secondly, our study design was cross-sectional and the main conclusions were made from correlational analyses. Therefore, longitudinal studies with larger sample size should be more reward to explore the causality between cerebral blood supply, brain structural damage and cognitive outcome. Thirdly, we could not make sure all the AD patients have been precluded. In fact, combined involvement of AD-type and vascular pathology were commonly seen in elderly people with cognitive decline (Neuropathology Group. Medical Research Council Cognitive Function and Aging Study, 2001). Lastly, the global cognitive function was merely assessed by most extensively used MMSE score, which may not be sensitive enough for detecting mild cognitive impairment.

## Conclusion

Cerebral perfusion status may have a significant impact on the maintenance of white matter health in subjects with leukoaraiosis. Meanwhile, cortex alterations along with WMH may mainly secondary to remote white matter degeneration other than hypoperfusion. Global cognitive outcome was more strongly associated with white matter integrity than with blood supply. DTI parameters, especially FA could serve as a potent imaging indicator for detecting the invisible disruption of white matter microarchitecture and implying its potential cognitive sequelae.

## Author Contributions

GZ drafted and revised the manuscript, participated in study concept and design, conducted the statistical analyses, analyzed and interpreted the data. ML participated in study concept and design, data interpretation and made a major contribution in revising the manuscript. RZ participated in the study design and made contribution in revising the manuscript. YJ, XY, and YZ assisted in designing the MRI sequences and imaging analysis. CL assessed the cognitive function of the participants. LT made contribution in revising the manuscript. LL validated the statistical analyses and made contribution in revising the manuscript.

## Conflict of Interest Statement

The authors declare that the research was conducted in the absence of any commercial or financial relationships that could be construed as a potential conflict of interest.

## Footnotes

1 http://www.fmrib.ox.ac.uk/fsl

2 https://www.nitrc.org/projects/dke/

3 www.itksnap.org

## Supplementary Material

FIGURE S1 Multimodal MRI images from a typical subject with leukoaraiosis: **(A)** FLAIR image; **(B)** WMH mask (red); **(C)** NAWM mask (red); **(D)** Cortex mask (red); **(E–H)** were the co-registered corresponding FA, MD, MK and CBF images, respectively. FLAIR, fluid attenuated inversion recovery; WMH, white matter hyperintensities; NAWM, normal-appearing white matter; FA, fractional anisotropy. MD, mean diffusivity; MK, mean kurtosis; CBF, cerebral blood flow.

FIGURE S2 **(A–H)** Scatter plots of mini-mental state examination (MMSE) score in relations to WMH-FA **(A)**, NAWM-FA **(B)**, WMH-MD **(C)**, NAWM-MD **(D)**, WMH-CBF **(E)**, NAWM-CBF **(F)**, Cortex-CBF **(G)**, and Cortex-MK^#^ **(H)**, respectively. ^#^Indicates subset analysis.
