# Supplementary figures and images for: Better Correlation of Cognitive Function to White Matter Integrity than to Blood Supply in Subjects with Leukoaraiosis
Source: Front Aging Neurosci. 2017 Jun 12;9:185. doi: 10.3389/fnagi.2017.00185 (PMC5466957; doi:10.3389/fnagi.2017.00185)

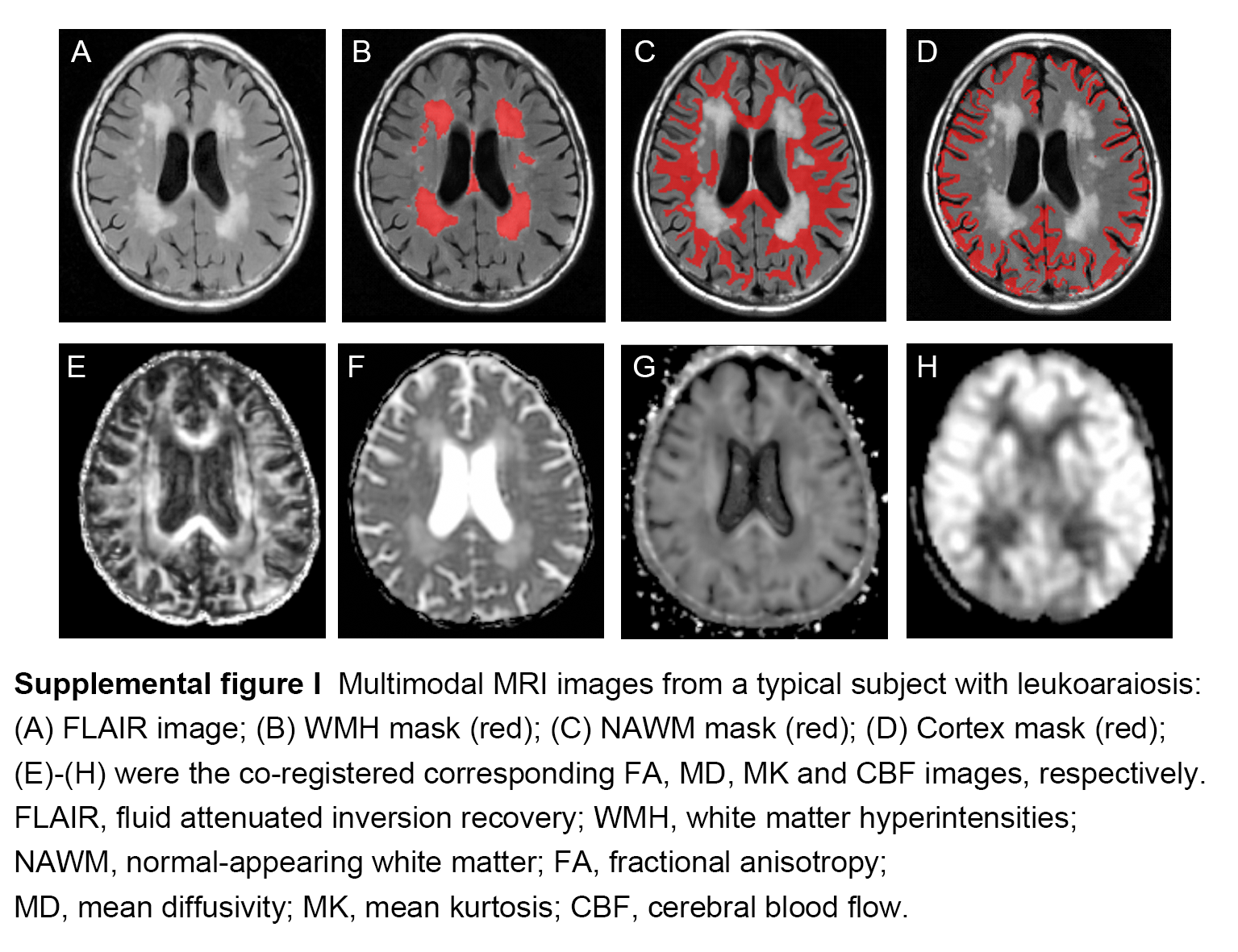

Supplement: FIGURE S1 — Multimodal MRI images from a typical subject with leukoaraiosis: (A) FLAIR image; (B) WMH mask (red); (C) NAWM mask (red); (D) Cortex mask (red); (E–H) were the co-registered corresponding FA, MD, MK and CBF images, respectively. FLAIR, fluid attenuated inversion recovery; WMH, white matter hyperintensities; NAWM, normal-appearing white matter; FA, fractional anisotropy. MD, mean diffusivity; MK, mean kurtosis; CBF, cerebral blood flow. [file Image_1.tif]

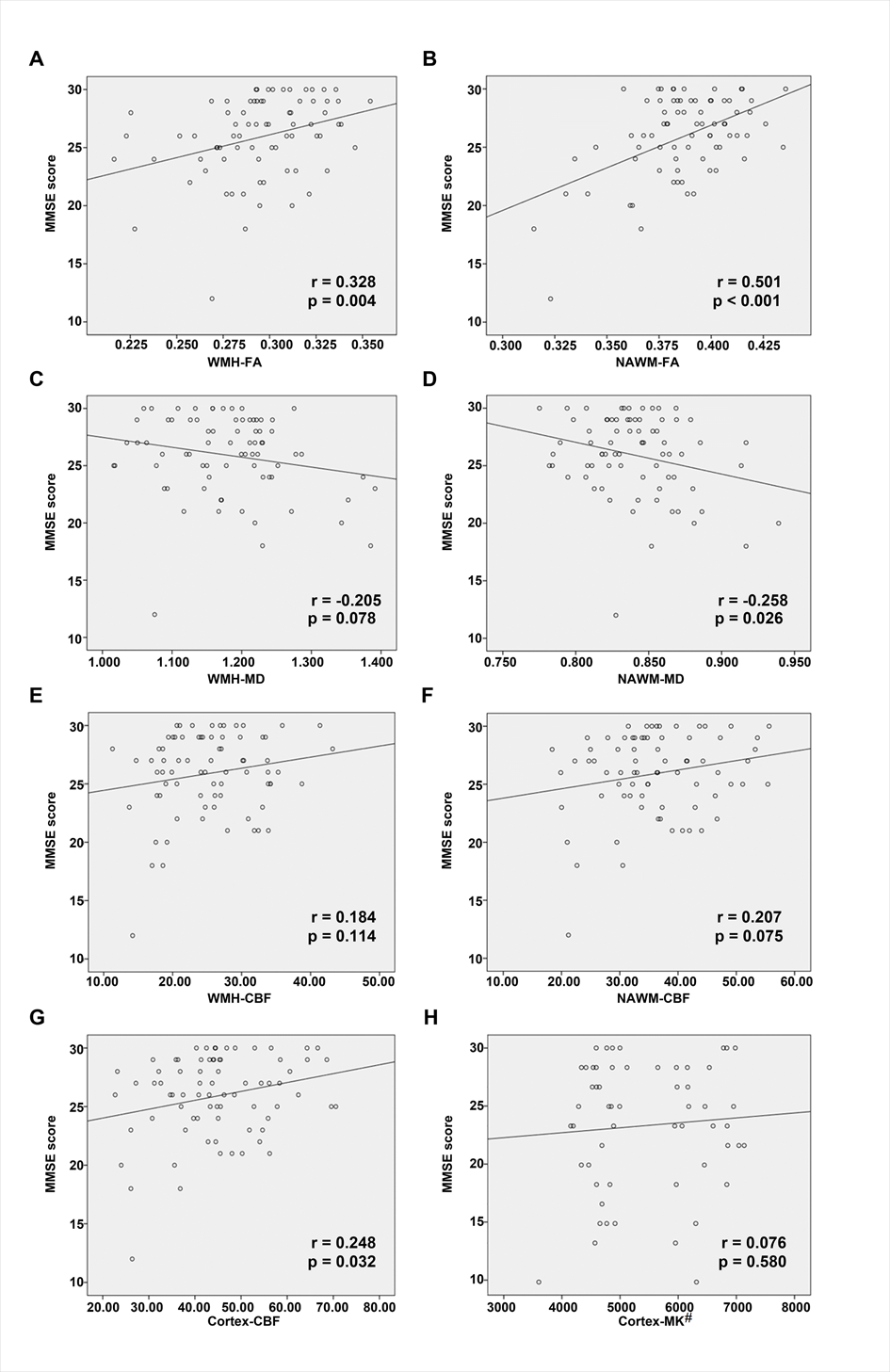

Supplement: FIGURE S2 — (A–H) Scatter plots of mini-mental state examination (MMSE) score in relations to WMH-FA (A), NAWM-FA (B), WMH-MD (C), NAWM-MD (D), WMH-CBF (E), NAWM-CBF (F), Cortex-CBF (G), and Cortex-MK# (H), respectively. #Indicates subset analysis. [file Image_2.TIF]
